# Crystal structure, Hirshfeld surface analysis and DFT studies of (*E*)-2-{[(3-chloro-4-methyl­phen­yl)imino]­meth­yl}-4-methyl­phenol

**DOI:** 10.1107/S2056989020009421

**Published:** 2020-07-21

**Authors:** Md. Serajul Haque Faizi, Emine Berrin Cinar, Alev Sema Aydin, Erbil Agar, Necmi Dege, Ashraf Mashrai

**Affiliations:** a PG Department of Chemistry, Langat Singh College, B. R. A. Bihar University, Muzaffarpur, Bihar 842001, India; b Ondokuz Mayıs University, Faculty of Arts and Sciences, Department of Physics, 55139, Samsun, Turkey; c Ondokuz Mayıs University, Faculty of Arts and Sciences, Department of Chemistry, 55139, Samsun, Turkey; d Department of Pharmacy, University of Science and Technology, Ibb Branch, Ibb, Yemen

**Keywords:** crystal structure, 3-chloro-4-methyl­aniline, 2-hy­droxy-5-methyl­benzaldehyde, Schiff base

## Abstract

The title compound, C_15_H_14_ClNO, was synthesized by condensation reaction of 2-hy­droxy-5-methyl­benzaldehyde and 3-chloro-4-methyl­aniline, and crystallizes in the monoclinic space group *P*2_1_/c. The 3-chloro­benzene ring is inclined to the phenol ring by 9.38 (11)°. The configuration about the C=N bond is *E* and an intra­molecular O—H⋯N hydrogen bond forms an *S*(6) ring motif. The Hirshfeld surface analysis of the crystal structure indicates that the most important contributions for the packing arrangement are from H⋯H (43.8%) and C⋯H/H⋯C (26.7%) inter­actions. The density functional theory (DFT) optimized structure at the B3LYP/ 6–311 G(d,p) level is compared with the experimentally determined mol­ecular structure and the HOMO–LUMO energy gap is provided.

## Abstract 2

In the title Schiff base compound, the hy­droxy group forms a intra­molecular hydrogen bond to the imine *N* atom generating an *S*(6) ring motif. The 3-chloro­benzene ring is inclined to the phenol ring by 9.38 (11)°. The configuration about the C=N bond is *E*.

## Chemical context   

Schiff bases contain the azomethine moiety (–*R*CH=N–*R*′) and are prepared by condensation reactions between amines and active carbonyl compounds. Schiff bases are employed as catalyst carriers (Grigoras *et al.*, 2001 ▸), thermo-stable mater­ials (Vančo *et al.*, 2004 ▸), metal–cation complexing agents and in biological systems (Taggi *et al.*, 2002 ▸). Schiff bases show biological activities including anti­bacterial, anti­fungal, anti­cancer, anti­viral and herbicidal activities (Desai *et al.*, 2001 ▸; Singh & Dash, 1988 ▸; Karia & Parsania, 1999 ▸; Siddiqui *et al.*, 2006 ▸). Moreover, Schiff base ligands are potentially capable of forming stable complexes by coordination of metal ions with their nitro­gen atoms as donors (Ebrahimipour *et al.*, 2012 ▸). They are important for their photochromic properties and have applications in various fields such as the measurement and control of radiation intensities in imaging systems, optical computers, electronics, optoelectronics and photonics (Iwan *et al.*, 2007 ▸). The present work is a part of an ongoing structural study of Schiff bases and their utilization in the synthesis of quinoxaline derivatives (Faizi *et al.*, 2018 ▸), fluorescence sensors (Faizi *et al.*, 2016 ▸; Mukherjee *et al.*, 2018 ▸; Kumar *et al.*, 2017 ▸, 2018 ▸) and non-linear optical properties (Faizi *et al.*, 2020 ▸). We report herein on the synthesis (from 2-hy­droxy-5-methyl­benzaldehyde and 3-chloro-4-methyl­aniline), crystal structure, Hirshfeld surface analysis and DFT computational calculations of the title compound, (I). The results of calculations by density functional theory (DFT) carried out at the B3LYP/6–311 G(d,p) level are compared with the experimentally determined mol­ecular structure in the solid state.
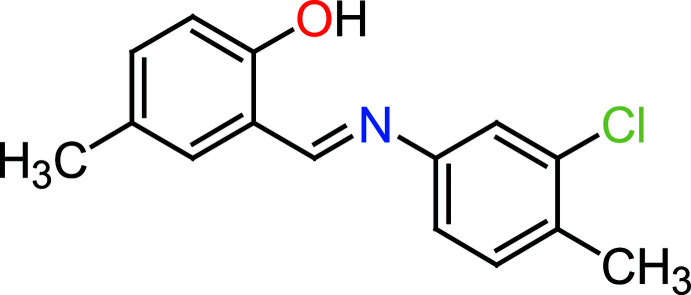


## Structural commentary   

**Figure 1:**
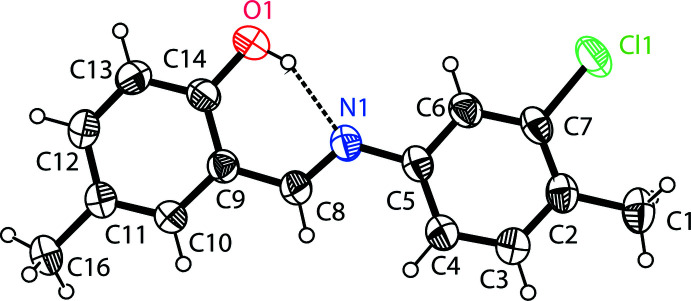
The mol­ecular structure of the title compound (I), showing the atom labelling and the inter­molecular O—H⋯N hydrogen bond as a dashed line. Displacement ellipsoids are drawn at the 40% probability level.

The mol­ecular structure of the title compound (I) is shown in Fig. 1 ▸. An intra­molecular O—H⋯N hydrogen bond is observed (Table 1 ▸ and Fig. 1 ▸). This is a relatively common feature in analogous imine–phenol compounds (see *Database survey* section). The imine group, which displays a C9—C8— N1—C5 torsion angle of −177.49 (18)°, contributes to the general non-planarity of the mol­ecule. The chloro­benzene ring (C2–C7) is inclined by 9.38 (11)° to the phenol ring (C9–C14). The configuration of the C7=N1 bond of this Schiff base is *E*, and the intra­molecular O1—H1⋯N1 hydrogen bond forms an *S*(6) ring motif (Fig. 1 ▸ *a* and Table 1 ▸). The C14—O1 distance [1.354 (2) Å] is close to normal values reported for single C—O bonds in phenols and salicyl­idene­amines (Ozeryanskii *et al.*, 2006 ▸). The N1—C8 bond is short at 1.281 (3) Å, indicating the existence of an imine bond, while the long C8—C9 bond [1.446 (3) Å] implies a single bond. All these data support the existence of the phenol–imine tautomer for (I) in its crystalline state. These features are similar to those observed in related 4-di­methyl­amino-*N*-salicylideneanilines (Wozniak *et al.*, 1995 ▸; Pizzala *et al.*, 2000 ▸). The C—N, C=N and C—C bond lengths are normal and close to the values observed in related structures (Faizi *et al.*, 2017 ▸).

## Supra­molecular features   

**Figure 2:**
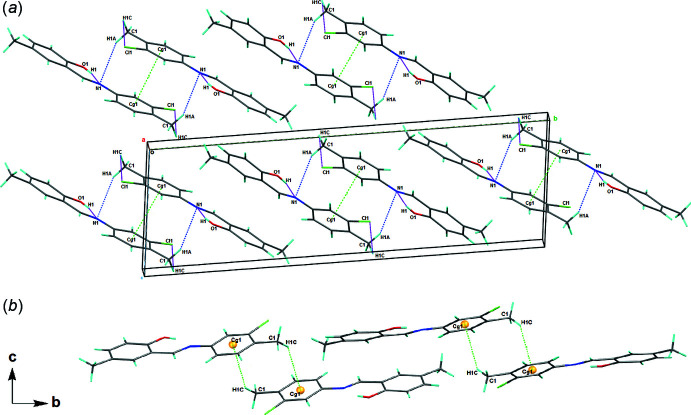
A view along the *a* axis of the crystal packing of title compound (I) showing (*a*) the C1—H1*C*⋯*Cg*1 inter­actions and (*b*) the most important inter­actions as dashed lines.

In the crystal packing of (I), the mol­ecules are linked by C1—H1*A*⋯N1 [H1*A*⋯N1(−*x* + 1, −*y* + 1, −*z* + 1) = 2.86 Å] inter­actions, forming sheets propagating along the *a*-axis direction (Fig. 2 ▸ *a*). Weak C—H⋯π inter­actions [C1—H1*C*⋯*Cg*1(−*x*, −*y* + 2, −*z*) = 2.92 Å] are observed (Table 1 ▸ and Fig. 2 ▸ *b*). Notably, weak π–π stacking inter­actions between chloro­benzene rings [*Cg*1⋯*Cg*1(−*x* + 1, −*y* + 1, −*z* + 1) = 3.7890 (2) Å, where *Cg*1 is the centroid of the C2–C7 ring] along the *a* axis lead to the formation of a three-dimensional network.

## Hirshfeld surface analysis   

**Figure 3:**
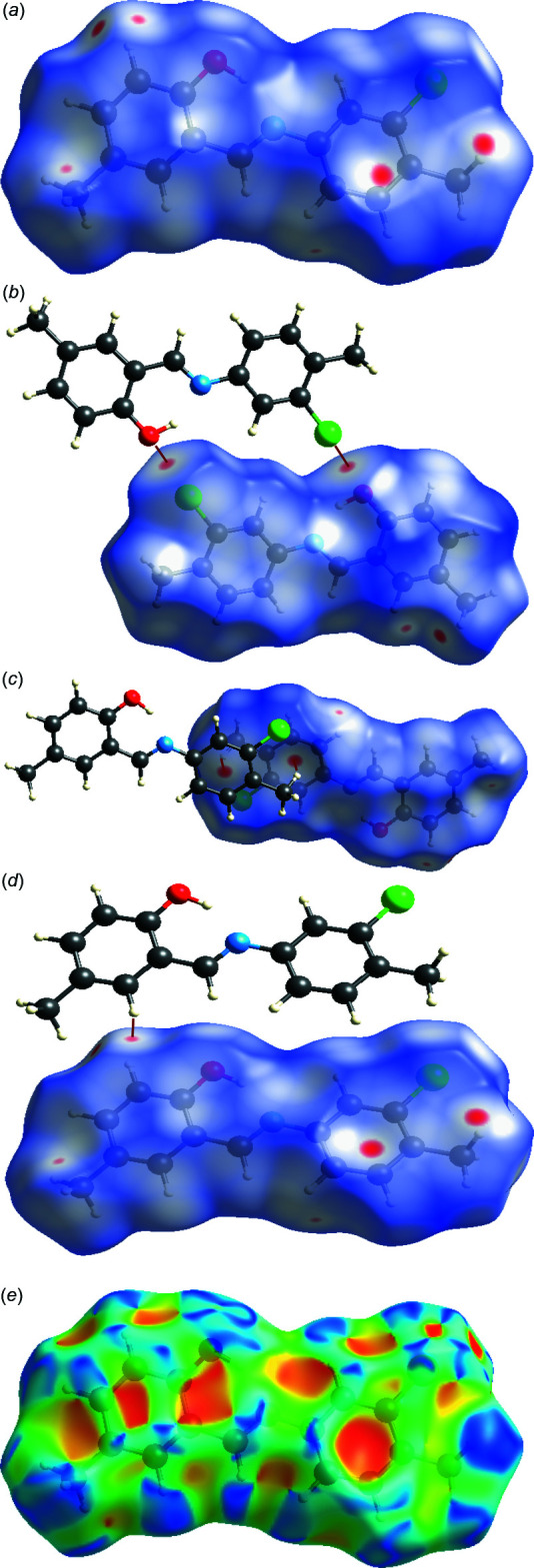
A view of the three-dimensional Hirshfeld surface for (I), plotted over (*a*)–(*d*) *d*_norm_ and (*e*) shape-index.

The inter­molecular inter­actions were investigated qu­anti­tatively and visualized with *Crystal Explorer 17.5* (Turner *et al.*, 2017 ▸; Spackman *et al.*, 2009 ▸). The shorter and longer contacts are indicated as red and blue spots, respectively, on the Hirshfeld surfaces, and contacts with distances approximately equal to the sum of the van der Waals radii are represented as white spots. The *d*_norm_ (*a*–*d*) and shape index (*e*) surface mappings are shown in Fig. 3 ▸. The most important red spots on the *d*_norm_ surface represent O1⋯Cl1 inter­actions (Fig. 3 ▸ *b*) and C1—H1*C*⋯*Cg*1 inter­actions (Fig. 3 ▸ *c*). Some additional inter­actions indicated by light-red spots are corresponding to contacts around phenolic and chloro­benzene rings (Fig. 3 ▸ *d*). The red and blue triangles are absent on the shape-index surface, which indicates there are no strong *π–π* stacking inter­actions in the crystal structure.

**Figure 4:**
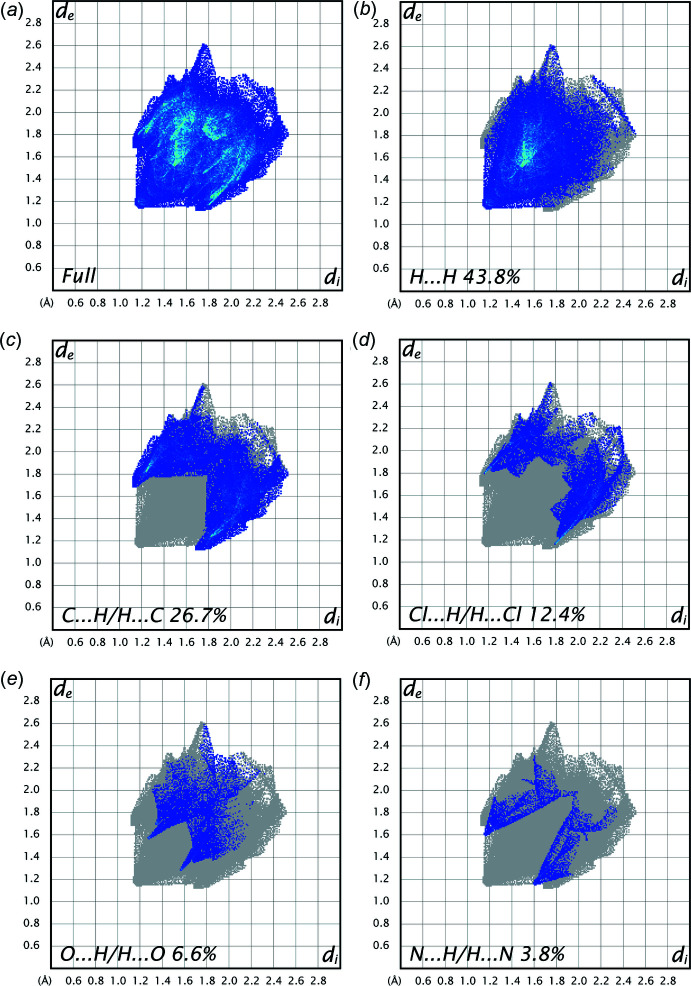
(*a*) The overall two-dimensional fingerprint plot for the title compound and (*b*)–(*f*) those delineated into H⋯H, C⋯H/H⋯C, Cl⋯H/H⋯Cl, O⋯H/H⋯O and N⋯H/H⋯N contacts, respectively.

Analysis of the two-dimensional fingerprint plots (Fig. 4 ▸ *a*–*f*) indicates that the H⋯H (43.8%) inter­actions are the major factor in the crystal packing with C⋯H/H⋯C (26.7%) inter­actions making the next highest contribution. The percentage contributions of other weak inter­actions are: Cl⋯H/H⋯Cl (12.4%), O⋯H/H⋯O (6.6%) and N⋯H/H⋯N (3.8%).

## DFT calculations   

**Figure 5:**
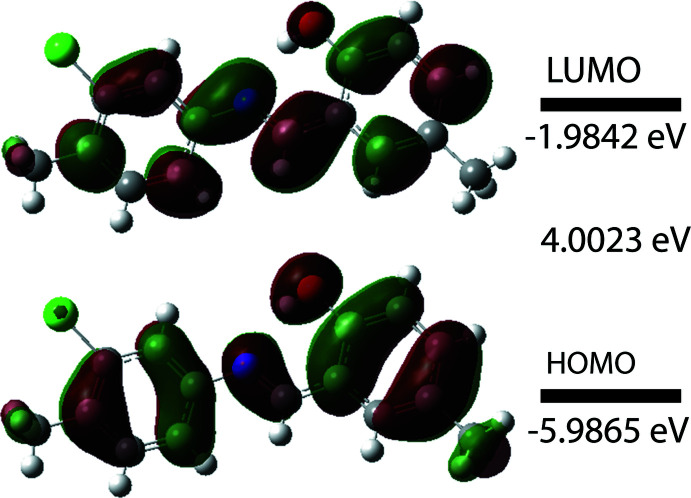
Mol­ecular orbitals showing the HOMO–LUMO electronic transition in the title compound.

The optimized structure in the gas phase of compound (I) was generated theoretically *via* density functional theory (DFT) using standard B3LYP functional and 6–311 G(d,p) basis-set calculations (Becke, 1993 ▸) as implemented in *GAUSSIAN 09* (Frisch *et al.*, 2009 ▸). The theoretical and experimental results are in good agreement (Table 2 ▸). The highest-occupied mol­ecular orbital (HOMO), acting as an electron donor, and the lowest-unoccupied mol­ecular orbital (LUMO), acting as an electron acceptor, are very important parameters for quantum chemistry. When the energy gap is small, the mol­ecule is highly polarizable and has high chemical reactivity (Fukui, 1982 ▸; Khan *et al.*, 2015 ▸). The DFT calculations provide some important information on the reactivity and site selectivity of the mol­ecular framework, *E*_HOMO_ and *E*_LUMO_, which clarify the inevitable charge-exchange collaboration inside the studied material, electronegativity (χ), hardness (η), electrophilicity (ω), softness (*σ*) and fraction of electron transferred (*ΔN*). These data are recorded in Table 3 ▸. The significance of η and *σ* is for the evaluation of both the reactivity and stability. The electron transition from the HOMO to the LUMO energy level is shown in Fig. 5 ▸. The HOMO and LUMO are localized in the plane extending from the whole 2-{[(3-chloro-4-methyl­phen­yl)imino]­meth­yl}-4-methyl­phenol ring. The energy band gap [Δ*E* = *E*_LUMO_ − *E*_HOMO_] of the mol­ecule is 4.0023 eV, the frontier mol­ecular orbital energies *E*_HOMO_ and *E*_LUMO_ being −5.9865 eV and −1.9842 eV, respectively. The dipole moment of (I) is estimated to be 4.30 Debye.

## Database survey   

A search of the Cambridge Structural Database (CSD, version 5.39; Groom *et al.*, 2016 ▸) gave 13 hits for the 2-{[(3-chloro-4-methyl­phen­yl)imino]­meth­yl}-4-methyl­phenol moiety. Out of 13, only a few are very closely related to the title compound. In (*E*)-4-meth­oxy-2-{[(4-methyl­phen­yl)imino]­meth­yl}phenol (DUPGOL; Koşar *et al.*, 2010 ▸), the methyl group is replaced by a meth­oxy group and the dihedral angle between the benzene rings is 5.46 (2)°. In 2-[(*E*)-(5-chloro-2-methyl­phen­yl)imino­meth­yl]-4-methyl­phenol (AFILAE; Zheng, 2013 ▸), the dihedral angle between the planes of the chloro­phenyl and methyl­phenol rings is 35.0 (3)°. In 2-{(*E*)-[(3-chloro-4-meth­yl­phen­yl)imino]­meth­yl}-4-(tri­fluoro­meth­oxy)phenol (TERTUI; Atalay *et al.*, 2017 ▸), the dihedral angle between the benzene rings is 8.3 (2)° and an intra­molecular O—H⋯N hydrogen bond closes an *S*(6) ring. In 2-{(*E*)-[(3-iodo-4-methyl­phen­yl)imino]­meth­yl}-4-(tri­fluoro­meth­oxy)phenol (XEBCOY; Pekdemir *et al.*, 2012 ▸), the dihedral angle between the two benzene rings is 12.4 (2)°. For 4-[(2-hy­droxy-5-meth­oxy­benzyl­idene)amino]­benzo­nitrile (XIGNEI; Chiang *et al.*, 2013 ▸), a complex with zinc is reported. In *N*-(5-hy­droxy­salicyl­idene)-2,4,6-tri­methyl­aniline (ZIKNOW; Tenon *et al.*, 1995 ▸), the angle between the planes of the benzene rings is 74.5 (1)° and chlorine is absent.

## Synthesis and crystallization   

The title compound was prepared by refluxing mixed solutions of 2-hy­droxy-5-methyl­benzaldehyde (34.0 mg, 0.25 mmol) in ethanol (15 ml) and 3-chloro-4-methyl­aniline (35.4 mg, 0.25 mmol) in ethanol (15 ml). The reaction mixture was stirred for 5 h under reflux. Single crystals of the title compound suitable for X-ray analysis were obtained by slow evaporation of an ethanol solution (yield 65%, m.p. 383–386 K).

## Refinement   

Crystal data, data collection and structure refinement details are summarized in Table 4 ▸. The hy­droxy *H* atom was located in a difference-Fourier map and its positional parameters were refined freely with *U*_iso_(H) = 1.5*U*_eq_(O). Other H atoms were fixed geometrically and treated as riding with C—H = 0.96 Å (meth­yl) or 0.93 Å (aromatic), and *U*_iso_(H) = 1.2*U*_eq_(C) for aromatic *H* atoms or *U*_iso_(H) = 1.5*U*_eq_(C) for methyl H atoms.

## Supplementary Material

Crystal structure: contains datablock(s) I. DOI: 10.1107/S2056989020009421/vm2236sup1.cif

Structure factors: contains datablock(s) I. DOI: 10.1107/S2056989020009421/vm2236Isup2.hkl

Supporting information file. DOI: 10.1107/S2056989020009421/vm2236Isup3.cml

CCDC reference: 2015356

Additional supporting information:  crystallographic information; 3D view; checkCIF report

## Figures and Tables

**Table 1:** Hydrogen-bond geometry (Å, °) *Cg*1 is the centroid of the C2–C7 ring.

*D*—H⋯*A*	*D*—H	H⋯*A*	*D*⋯*A*	*D*—H⋯*A*
O1—H1⋯N1	0.79 (4)	1.89 (3)	2.625 (3)	153 (3)
C1—H1*C*⋯Cl1	0.96	2.91	3.072 (3)	91
C1—H1*A*⋯N1^i^	0.96	2.86	3.734 (3)	152
C1—H1*C*⋯*Cg*1^ii^	0.96	2.92	3.617 (2)	131

Symmetry codes: (i) 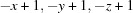; (ii) 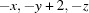.

**Table 2:** Comparison of observed (X-ray data) and calculated (DFT) geometric parameters (Å, °)

Parameter	X-ray	B3LYP/6–311G(d,p)
O1—C14	1.354 (2)	1.354
C7—Cl1	1.735 (2)	1.735
N1—C8	1.281 (3)	1.281
C8—C9	1.446 (3)	1.446
N1—C5	1.418 (3)	1.418
C2—C7	1.385 (3)	1.385
C13—C14—C9	119.36 (19)	119.4
C9—C8—N1	121.82 (19)	121.8
C8—N1—C5	122.08 (19)	122.1

**Table 3:** Calculated mol­ecular energies for (I)

Mol­ecular Energy (a.u.) (eV)	Compound (I)
Total Energy *TE* (eV)	−31841.0844
*E*_HOMO_ (eV)	−5.9865
*E*_LUMO_ (eV)	−1.9842
Gap, *ΔE* (eV)	4.0023
Dipole moment, *μ* (Debye)	4.30
Ionization potential, *I* (eV)	5.9865
Electron affinity, *A*	1.9842
Electronegativity, *χ*	3.985
Hardness, *η*	2.001
Electrophilicity index, *ω*	3.968
Softness, *σ*	0.250
Fraction of electron transferred, *ΔN*	0.754

**Table 4:** Experimental details

Crystal data	
Chemical formula	C_15_H_14_ClNO
*M*_r_	259.72
Crystal system, space group	Monoclinic, *P*2_1_/*c*
Temperature (K)	296
*a*, *b*, *c* (Å)	8.0534 (5), 6.3764 (3), 25.3657 (16)
β (°)	96.392 (5)
*V* (Å^3^)	1294.47 (13)
*Z*	4
Radiation type	Mo *K*α
μ (mm^−1^)	0.28
Crystal size (mm)	0.65 × 0.37 × 0.21

Data collection	
Diffractometer	Stoe IPDS 2
Absorption correction	Integration (*X-RED32*; Stoe & Cie, 2002 ▸)
*T*_min_, *T*_max_	0.885, 0.958
No. of measured, independent and observed [*I* > 2σ(*I*)] reflections	7752, 2414, 1801
*R*_int_	0.040
(sin θ/λ)_max_ (Å^−1^)	0.606

Refinement	
*R*[*F* ^2^ > 2σ(*F* ^2^)], *wR*(*F* ^2^), *S*	0.045, 0.137, 1.03
No. of reflections	2414
No. of parameters	169
H-atom treatment	H atoms treated by a mixture of independent and constrained refinement
Δρ_max_, Δρ_min_ (e Å^−3^)	0.22, −0.26

Computer programs: *X-AREA* and *X-SHAPE* (Stoe & Cie, 2002 ▸), *SHELXT2018/2* (Sheldrick, 2015*a* ▸), *SHELXL2018/3* (Sheldrick, 2015*b* ▸), *ORTEP-3 for Windows* (Farrugia, 2012 ▸) and *XP* in *SHELXTL* (Sheldrick, 2008 ▸).

## supplementary crystallographic information

### Crystal data

**Table:**

C_15_H_14_ClNO	*F*(000) = 544
*M**_r_* = 259.72	*D*_x_ = 1.333 Mg m^−^^3^
Monoclinic, *P*2_1_/*c*	Mo *K*α radiation, λ = 0.71073 Å
*a* = 8.0534 (5) Å	Cell parameters from 9569 reflections
*b* = 6.3764 (3) Å	θ = 1.6–30.3°
*c* = 25.3657 (16) Å	µ = 0.28 mm^−^^1^
β = 96.392 (5)°	*T* = 296 K
*V* = 1294.47 (13) Å^3^	Stick, orange
*Z* = 4	0.65 × 0.37 × 0.21 mm

### Data collection

**Table:**

Stoe IPDS 2 diffractometer	2414 independent reflections
Radiation source: sealed X-ray tube, 12 x 0.4 mm long-fine focus	1801 reflections with *I* > 2σ(*I*)
Plane graphite monochromator	*R*_int_ = 0.040
Detector resolution: 6.67 pixels mm^-1^	θ_max_ = 25.5°, θ_min_ = 1.6°
rotation method scans	*h* = −9→9
Absorption correction: integration (X-RED32; Stoe & Cie, 2002)	*k* = −7→7
*T*_min_ = 0.885, *T*_max_ = 0.958	*l* = −30→30
7752 measured reflections	

### Refinement

**Table:**

Refinement on *F*^2^	0 restraints
Least-squares matrix: full	Hydrogen site location: mixed
*R*[*F*^2^ > 2σ(*F*^2^)] = 0.045	H atoms treated by a mixture of independent and constrained refinement
*wR*(*F*^2^) = 0.137	*w* = 1/[σ^2^(*F*_o_^2^) + (0.0845*P*)^2^ + 0.059*P*] where *P* = (*F*_o_^2^ + 2*F*_c_^2^)/3
*S* = 1.03	(Δ/σ)_max_ < 0.001
2414 reflections	Δρ_max_ = 0.22 e Å^−^^3^
169 parameters	Δρ_min_ = −0.26 e Å^−^^3^

### Special details

**Table:**

Geometry. All esds (except the esd in the dihedral angle between two l.s. planes) are estimated using the full covariance matrix. The cell esds are taken into account individually in the estimation of esds in distances, angles and torsion angles; correlations between esds in cell parameters are only used when they are defined by crystal symmetry. An approximate (isotropic) treatment of cell esds is used for estimating esds involving l.s. planes.

### Fractional atomic coordinates and isotropic or equivalent isotropic displacement parameters (Å^2^)

**Table:**

	*x*	*y*	*z*	*U*_iso_*/*U*_eq_	
Cl1	0.74526 (10)	0.21146 (12)	0.56404 (2)	0.0820 (3)	
O1	0.3423 (3)	0.0128 (3)	0.32974 (7)	0.0729 (5)	
N1	0.5050 (2)	0.3352 (3)	0.37421 (7)	0.0546 (4)	
C9	0.3492 (2)	0.3538 (3)	0.28875 (8)	0.0495 (5)	
C5	0.6021 (3)	0.4244 (3)	0.41869 (8)	0.0517 (5)	
C10	0.2953 (3)	0.4790 (3)	0.24498 (8)	0.0532 (5)	
H10	0.333321	0.616621	0.244352	0.064*	
C6	0.6276 (3)	0.2994 (3)	0.46336 (8)	0.0558 (5)	
H6	0.582700	0.164970	0.462883	0.067*	
C8	0.4537 (3)	0.4436 (3)	0.33310 (8)	0.0540 (5)	
H8	0.484407	0.583899	0.331729	0.065*	
C7	0.7199 (3)	0.3742 (4)	0.50893 (8)	0.0560 (5)	
C14	0.2942 (3)	0.1445 (3)	0.28904 (8)	0.0537 (5)	
C11	0.1881 (3)	0.4072 (3)	0.20271 (8)	0.0544 (5)	
C2	0.7902 (3)	0.5727 (4)	0.51180 (8)	0.0569 (5)	
C3	0.7630 (3)	0.6946 (4)	0.46627 (9)	0.0624 (6)	
H3	0.808124	0.828887	0.466715	0.075*	
C4	0.6720 (3)	0.6247 (4)	0.42059 (9)	0.0606 (6)	
H4	0.657040	0.711090	0.390901	0.073*	
C12	0.1343 (3)	0.1995 (4)	0.20491 (9)	0.0603 (5)	
H12	0.061070	0.146941	0.177099	0.072*	
C13	0.1860 (3)	0.0705 (4)	0.24686 (9)	0.0618 (6)	
H13	0.148288	−0.067273	0.246965	0.074*	
C15	0.1266 (3)	0.5478 (4)	0.15702 (9)	0.0699 (7)	
H15A	0.110835	0.687133	0.169929	0.105*	
H15B	0.022293	0.495293	0.140088	0.105*	
H15C	0.207326	0.550746	0.131900	0.105*	
C1	0.8890 (3)	0.6569 (4)	0.56097 (9)	0.0722 (7)	
H1A	0.817191	0.672248	0.588488	0.108*	
H1B	0.935229	0.790941	0.553389	0.108*	
H1C	0.977895	0.561373	0.572449	0.108*	
H1	0.399 (4)	0.082 (5)	0.3505 (14)	0.099 (12)*	

### Atomic displacement parameters (Å^2^)

**Table:**

	*U*^11^	*U*^22^	*U*^33^	*U*^12^	*U*^13^	*U*^23^
Cl1	0.1001 (5)	0.0819 (5)	0.0596 (4)	0.0149 (4)	−0.0115 (3)	0.0193 (3)
O1	0.0944 (13)	0.0595 (10)	0.0617 (10)	−0.0062 (9)	−0.0051 (9)	0.0144 (8)
N1	0.0546 (10)	0.0596 (11)	0.0481 (9)	0.0028 (8)	−0.0013 (7)	0.0010 (8)
C9	0.0467 (11)	0.0521 (11)	0.0489 (11)	0.0015 (9)	0.0018 (8)	0.0018 (8)
C5	0.0497 (11)	0.0586 (12)	0.0458 (10)	0.0081 (9)	0.0014 (8)	0.0017 (8)
C10	0.0564 (12)	0.0506 (11)	0.0514 (11)	−0.0007 (9)	0.0007 (9)	0.0043 (9)
C6	0.0575 (12)	0.0535 (12)	0.0553 (11)	0.0105 (10)	0.0008 (9)	0.0040 (9)
C8	0.0542 (12)	0.0529 (12)	0.0528 (11)	0.0025 (9)	−0.0034 (9)	0.0023 (9)
C7	0.0569 (12)	0.0617 (13)	0.0481 (11)	0.0185 (10)	−0.0001 (9)	0.0050 (9)
C14	0.0601 (13)	0.0513 (11)	0.0496 (11)	0.0027 (10)	0.0062 (9)	0.0060 (9)
C11	0.0532 (12)	0.0616 (12)	0.0477 (11)	0.0017 (10)	0.0021 (9)	0.0008 (9)
C2	0.0531 (12)	0.0654 (14)	0.0510 (11)	0.0124 (10)	0.0010 (9)	−0.0064 (10)
C3	0.0648 (14)	0.0625 (14)	0.0583 (13)	−0.0030 (11)	0.0000 (10)	−0.0016 (10)
C4	0.0646 (13)	0.0642 (13)	0.0515 (11)	−0.0047 (11)	−0.0003 (10)	0.0060 (10)
C12	0.0586 (12)	0.0700 (14)	0.0507 (11)	−0.0053 (11)	−0.0003 (9)	−0.0081 (10)
C13	0.0692 (14)	0.0540 (12)	0.0621 (13)	−0.0097 (11)	0.0067 (11)	−0.0030 (10)
C15	0.0756 (16)	0.0780 (17)	0.0524 (12)	0.0013 (13)	−0.0093 (11)	0.0077 (11)
C1	0.0725 (15)	0.0864 (18)	0.0545 (13)	0.0117 (13)	−0.0067 (11)	−0.0140 (12)

### Geometric parameters (Å, º)

**Table:**

Cl1—C7	1.735 (2)	C11—C12	1.397 (3)
O1—C14	1.354 (2)	C11—C15	1.505 (3)
O1—H1	0.79 (4)	C2—C3	1.389 (3)
N1—C8	1.281 (3)	C2—C1	1.502 (3)
N1—C5	1.418 (3)	C3—C4	1.374 (3)
C9—C10	1.397 (3)	C3—H3	0.9300
C9—C14	1.406 (3)	C4—H4	0.9300
C9—C8	1.446 (3)	C12—C13	1.372 (3)
C5—C6	1.382 (3)	C12—H12	0.9300
C5—C4	1.394 (3)	C13—H13	0.9300
C10—C11	1.378 (3)	C15—H15A	0.9600
C10—H10	0.9300	C15—H15B	0.9600
C6—C7	1.388 (3)	C15—H15C	0.9600
C6—H6	0.9300	C1—H1A	0.9600
C8—H8	0.9300	C1—H1B	0.9600
C7—C2	1.385 (3)	C1—H1C	0.9600
C14—C13	1.385 (3)		
			
C14—O1—H1	105 (2)	C7—C2—C1	123.1 (2)
C8—N1—C5	122.08 (19)	C3—C2—C1	120.7 (2)
C10—C9—C14	118.46 (18)	C4—C3—C2	122.6 (2)
C10—C9—C8	119.64 (19)	C4—C3—H3	118.7
C14—C9—C8	121.86 (18)	C2—C3—H3	118.7
C6—C5—C4	118.5 (2)	C3—C4—C5	120.1 (2)
C6—C5—N1	116.1 (2)	C3—C4—H4	120.0
C4—C5—N1	125.41 (19)	C5—C4—H4	120.0
C11—C10—C9	122.7 (2)	C13—C12—C11	122.0 (2)
C11—C10—H10	118.6	C13—C12—H12	119.0
C9—C10—H10	118.6	C11—C12—H12	119.0
C5—C6—C7	120.1 (2)	C12—C13—C14	120.4 (2)
C5—C6—H6	119.9	C12—C13—H13	119.8
C7—C6—H6	119.9	C14—C13—H13	119.8
N1—C8—C9	121.82 (19)	C11—C15—H15A	109.5
N1—C8—H8	119.1	C11—C15—H15B	109.5
C9—C8—H8	119.1	H15A—C15—H15B	109.5
C2—C7—C6	122.4 (2)	C11—C15—H15C	109.5
C2—C7—Cl1	119.58 (17)	H15A—C15—H15C	109.5
C6—C7—Cl1	118.04 (18)	H15B—C15—H15C	109.5
O1—C14—C13	118.7 (2)	C2—C1—H1A	109.5
O1—C14—C9	121.97 (19)	C2—C1—H1B	109.5
C13—C14—C9	119.36 (19)	H1A—C1—H1B	109.5
C10—C11—C12	117.03 (19)	C2—C1—H1C	109.5
C10—C11—C15	121.7 (2)	H1A—C1—H1C	109.5
C12—C11—C15	121.2 (2)	H1B—C1—H1C	109.5
C7—C2—C3	116.2 (2)		
			
C8—N1—C5—C6	170.44 (19)	C9—C10—C11—C15	177.6 (2)
C8—N1—C5—C4	−9.5 (3)	C6—C7—C2—C3	0.1 (3)
C14—C9—C10—C11	1.3 (3)	Cl1—C7—C2—C3	−179.31 (17)
C8—C9—C10—C11	−176.3 (2)	C6—C7—C2—C1	179.4 (2)
C4—C5—C6—C7	0.4 (3)	Cl1—C7—C2—C1	0.0 (3)
N1—C5—C6—C7	−179.51 (18)	C7—C2—C3—C4	−0.1 (3)
C5—N1—C8—C9	−177.49 (18)	C1—C2—C3—C4	−179.4 (2)
C10—C9—C8—N1	179.5 (2)	C2—C3—C4—C5	0.2 (4)
C14—C9—C8—N1	2.0 (3)	C6—C5—C4—C3	−0.4 (3)
C5—C6—C7—C2	−0.3 (3)	N1—C5—C4—C3	179.5 (2)
C5—C6—C7—Cl1	179.12 (16)	C10—C11—C12—C13	−0.6 (3)
C10—C9—C14—O1	179.3 (2)	C15—C11—C12—C13	−178.4 (2)
C8—C9—C14—O1	−3.1 (3)	C11—C12—C13—C14	0.4 (4)
C10—C9—C14—C13	−1.5 (3)	O1—C14—C13—C12	179.9 (2)
C8—C9—C14—C13	176.1 (2)	C9—C14—C13—C12	0.6 (3)
C9—C10—C11—C12	−0.3 (3)		

### Hydrogen-bond geometry (Å, º)

*Cg*1 is the centroid of the C2–C7 ring.

**Table:**

*D*—H···*A*	*D*—H	H···*A*	*D*···*A*	*D*—H···*A*
O1—H1···N1	0.79 (4)	1.89 (3)	2.625 (3)	153 (3)
C1—H1*C*···Cl1	0.96	2.91	3.072 (3)	91
C1—H1*A*···N1^i^	0.96	2.86	3.734 (3)	152
C1—H1*C*···*Cg*1^ii^	0.96	2.92	3.617 (2)	131

Symmetry codes: (i) −*x*+1, −*y*+1, −*z*+1; (ii) −*x*, −*y*+2, −*z*.
